# Dr. Kellogg on Aristocracy

**Published:** 1915-09

**Authors:** 


### The Department of Eugenics, Mental and Social Hygiene

CONDUCTED BY
DR. MALONE DUGGAN
813-814 Gibbs Building, San Antonio, Texas
All communications for this department should be sent to DR. DUGGAN

### Dr. Kellogg on Aristocracy.

At the same conference as above referred to, Dr. J. H. Kellogg, of Battle Creek, Michigan, said:

"The world needs a new aristocracy—a real aristocracy made up of Apollos and Venuses and their fortunate progeny. Instead of such an aristocracy, we are actually building up an aristocracy of lunatics, idiots, paupers and criminals. These unfit persons already have reached the proportions of a vast multitude—500,000 lunatics, 80,000 criminals, 100,000 paupers, 90,000 idiots, 90,000 epileptics, and we are supporting these defectives in idleness like real aristocrats, at an expense of one hundred million dollars a year, and this mighty host of mental and moral cripples is increasing, due to unrestricted marriage and other degenerative influences at a more rapid rate than the sounder part of the population, so that they are bound in time to constitute the majority unless some check is put on the increase."

This prophecy of the doctor's is not that of the alarmist but is a clear statement of the facts which society would do well to heed. Heretofore, society, imbued with the doctrine that environment alone was responsible, for character, leaving out of consideration the hereditary element, had built up a doctrine of humanitarianism that is responsible for the condition of which the doctor speaks. While environment is essential to the development of the organism, and while it in a large measure determines the direction which this development will take, it is absolutely limited by the possibilities of development inherent in the germ cell, which is the heredity of the organism. Therefore, as Mr. Burbanks says, heredity is "ten thousand times more important."

We take issue with the doctor, however, when he says that "we need an aristocracy made up of Apollos and Venuses." Race betterment cannot be attained by breeding any one kind of stock; but by the development of the best of all stocks so that the ultimate result will be a product that can best adjust itself to any and all conditions. What would be the result if we bred only race horses? There would then be no horses fitted for the plow or road. What would be the result, likewise, if we bred only Apollos and Venuses? There would be a race suited only for one environment.

By calling attention to this error in genetics, we do not mean to criticize the intent of the doctor's argument, for, on the whole, it is correct, but to clearly emphasize the important fact that the development of man is not the same as that of animals, and cannot be managed in the same way because of his social heredity. This marks the vast difference between the two and makes the development of man vastly more difficult.

